# Re: Is there a difference in the effect between the angiotensin‐converting enzyme inhibitor and angiotensin‐receptor blocker on the COVID‐19?

**DOI:** 10.1002/clc.23473

**Published:** 2020-11-12

**Authors:** Xiao Liu, Chuyan Long, Qinmei Xiong, Chen Chen, Jianyong Ma, Yuhao Su, Kui Hong

**Affiliations:** ^1^ Department of Cardiovascular Medicine The Second Affiliated Hospital of Nanchang University Jiangxi China; ^2^ Jiangxi Key Laboratory of Molecular Medicine Jiangxi China

To the Editor

We thank Dr Chen et al. for their insightful comments on our meta‐analysis and appreciate the opportunity to address their concerns. Dr Chen et al. suggested that a difference might exist between the effects of angiotensin‐converting enzyme inhibitors (ACEIs) and angiotensin‐receptor blockers (ARBs) on the COVID‐19. However, the limited studies precluded us from performing a subgroup analysis stratified by renin‐angiotensin‐aldosterone system inhibitors or blockers as described in our study limitations. Recently, several new articles were published, so we re‐searched the databases (Pubmed, Embase, medRxiv) and added three articles^1^, ^2^, ^3^ (combining with our pervious meta‐analysis^4^) for providing the specific results for a ACEIs or ARBs. As shown in Figure 1, there is no significant difference between ACEIs or ARB in the effect on positive COVID‐19, severity and death (*P* for positive COVID‐19 = .76, *P* for severe COVID‐19 = .63, *P* for death = .47) (Figure 1). Secondly, inflammation certainly plays a crucial role in COVID‐19. Another meta‐analysis also found that interleukin‐6 levels were reduced in the ACEI group COVID‐19 from positive patients vs non‐ACEIs.^5^ However, an open‐label study did not find a significant improvement of sarilumab (an interleukin‐6 blocker) in clinical improvement and mortality in patients with severe COVID‐19 compared with the standard of care group.^6^ Therefore, more trials were needed to clarify its mechanism of ACEIs/ARBs on immune response in COVID‐19.

**FIGURE 1:**
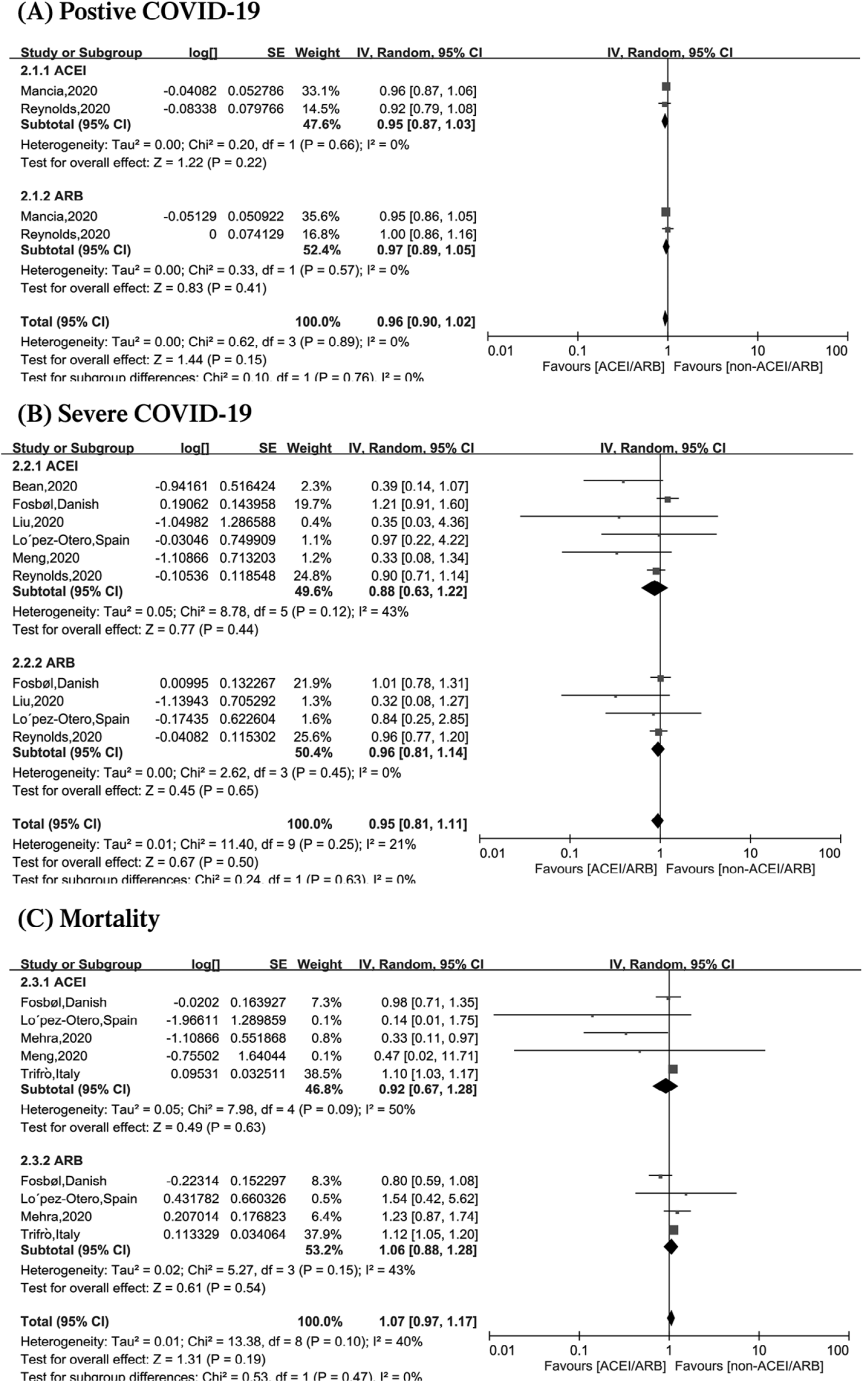
Summary of the associations between use of ACEI/ARB and clinical outcomes among patients with COVID‐19 stratified by ACEI and ARB: A, risk of COVID‐19 infection; B, risk of severe COVID‐19 infection; C, mortality. ACEI, angiotensin‐converting enzyme inhibitors; ARB, angiotensin‐receptor blockers

## CONFLICT OF INTEREST

The author declares no potential conflict of interest.

## Data Availability

Data available on request from the authors
